# The Response of Pathological Anatomy to the Question: "What Is Cancer?"

**Published:** 1847-10

**Authors:** 


### Art. VI.

*Den Pathologiske Anatomie's Svar paa Spörgsmaalet: Hoad er Cancer?* Ved Adolph Hannover, Lic. Med.—*Kjobenhavn*, 1843.

*The Response of Pathological Anatomy to the Question: "What is Cancer?"* By Adolph Hannover, Licentiate of Medicine. With a Lithographed Plate.—*Copenhagen*, 1843. pp. 106.

So much has recently appeared in the pages of this Journal, on the subject of Cancer, that we fear to exhaust the patience of our readers, when we present them with yet another essay on this important and obscure disease. Still we feel certain, that, in laying before the British public, the results arrived at by a laborious and conscientious investigator, by one, whose researches on respiration, and on the nervous system, have already earned for him a fair meed of fame; we are contributing to elucidate still further, a subject on which too much attention cannot be bestowed. Whether the work now before us tends to corroborate the researches of others, or to render doubtful some opinions which have been very generally adopted, we deem it of importance that our British readers should know what has been ascertained in regard to the pathology of cancer by a Danish writer, and how materially the investigations of Dr. Walshe are confirmed by the testimony of Dr. Hannover's experience. We shall confine ourselves, in this notice however, solely to the author's own opinions, only briefly adverting, where it is unavoidable, to the doctrines laid down, and defended by any other writer. Dr. Hannover divides his essay into three parts, in the first of which, he details minutely the anatomical and microscopical appearances of cancer; the succeeding part is devoted to the history of the disease, according to its locality in the various organs and parts of the human body; and the third and last comprises our author's own opinions on the origin and nature of the malady.

After a few remarks on the contradictory opinions, that have prevailed, regarding the true nature of cancer, Dr. Hannover proceeds to detail his own investigations with the microscope, into the phenomena of cancerous growth and formations.

It was not, as our author confesses, till he had overcome many difficulties, and experienced many grievous disappointments, that he decided upon rejecting the caudate cells, as a diagnostic sign of cancerous tissue, under the microscope, while, at the same time, he was enabled finally to determine, that another form of cell exhibiting several marked peculiarities, may really be regarded as characteristic of the presence of this malady. Such too we know to be the conclusion arrived at by Dr. Walshe, in regard at least to the value of the caudate cells, and from a close comparison of his description with those of Dr. Hannover, and especially with the lithographed plate given by the latter, we are inclined to believe that the *flat parietal opaque body* regarded by Dr. Walshe as a probable essential product of cancer, is to be found in the nucleus of the cancer-cell, described by our Danish investigator.

In imitation of Dr. Walshe, who was the first pathologist* to admit encephaloid, scirrhous and colloid, as three equally distinct species of one genus cancer, to the exclusion of melanosis and various other ill-defined products, Dr. Hannover recognises three species of cancerous degeneration: 1, the medullary (cancer medullaris); 2, the schirrous, (cancer schirrosus); and 3, colloid cancer (cancer alveolaris). He however hesitates, as to the propriety of associating this last with the two former, as he has never found in colloid cancer, those peculiar bodies, which he has invariably observed to be present in the two first-named species. But he acknowledges that he has had comparatively but few opportunities of investigating the microscopical appearances in colloid cancer.

In our author's definition of cancer we do not find much that is novel:

"Anatomically speaking, cancer may be defined as a heterologous organic formation, distinguished under the microscope by one peculiar feature, viz., a unique cell, which we will call a cancer-cell. In a pathological point of view, it may be described as a formation (depending probably on a peculiar diathesis), which destroys or replaces the normal structure of the part in which it occurs, having the tendency to be reproduced when the affected part is taken away, and in general giving rise to constitutional disorder and to cachexia, which eventually proves destructive to life."

Dr. Hannover, describes, as follows, the proper cancer-cells.

"These cells are round or oval, and only appear angular when closely pressed together. They are pale and finely granular on the surface, and form vesicles filled with a very pale fluid, as can best be shown by setting the whole mass in motion, when the cells may be observed to roll (*upon themselves?*). Their contour is never sharply defined. Within the walls of the cancer cells, we observe one or more nuclei, which proportionally are of considerable size. They appear somewhat darker than the containing cell, and are more sharply defined. We almost always observe several nuclei in one cancer-cell. These nuclei appear also to be hollow bodies. They are sometimes grooved in the centre, and exhibit a tendency to subdivision. The molecules of which the nucleus is composed cluster about the little, round, semitransparent nucleolus, of which one or two are to be found inclosed in each nucleus."

When cancer is softening, the membrane of which the cancer-cells are composed disappears altogether, and the nuclei alone remain, though not unchanged, for they have a shrunken withered appearance, and the nucleolus is no longer visible. In this form they bear the closest possible resemblance to pus-cells, but may easily be distinguished from these by the addition of a little acetic acid, which does not alter the withered aspect of the cancer nuclei, while in the cells of pus, it causes the immediate reappearance of the one, two, three, or four nucleoli. The above

* Cyclopædia of Surgery, July, 1840.

described cancer-cells have not been met with by Dr. Hannover in any part of the healthy body, nor in any diseased tissue, saving in that affected with cancer. The chief characteristic of these cells is the large nucleus, with a brilliant nucleolus, and the existence of several such nuclei in the same cells.

The other chief constituent (Hovedelementardeel) of cancer is cellular tissue, which is of course not confined solely to this morbid growth, nor does it appear there under any peculiar form.

"Under the microscope, we observe completely-formed cellular membrane, consisting of pale, cylindrical, branchless fibres, which are peculiarly recognisable by their serpentine course, and watered aspect (valtret udseende); and again we meet with cellular tissue in its embryonal form, i. e. as fibres springing from formative cells. The embryonal cells and fibres of cellular tissue appear under two forms: the former is that of pale, finely-granular cells, of very irregular shape, round, star-shaped, flat, or sometimes elliptical, or even still more prolonged. The nucleus is also extended, many nuclei occurring in one lengthened cell, but this last is formed by the junction lengthways (efter laengden) of several cells, by which means the nuclei are compressed so as to form a continuous thread or fibre. The nuclei are generally of a darker hue, and contain from one to four very small nucleoli. From either, or from both ends of those extended cells, there proceeds fibres which are finely granular, and certainly paler and broader than those of cellular tissue, but still the latter undoubtedly is formed by these prolongations of the cells."

These are the caudate cells, which have been regarded by Müller and Valentin, as almost, if not quite, characteristic of cancerous degeneration. The other species of fibre does not originate from cells, but from nuclei, which are very small spindle-shaped bodies, and destitute of nucleoli. Fine linear threads arise from one or both ends of these nuclei; they are somewhat thicker at their point of origin, and two often spring from the same nucleus. This latter form is but rarely seen in cancerous tissues.

The cancer-cells then, and cellular tissue, are the two chief constituents of the cancerous mass, but in addition to these, we likewise constantly meet with other substances, according to the parts of the body in which the morbid alteration may have taken place. Crystals, pus-cells, and exudation-corpuscles (Betaendeloeskugler) likewise occur as occasional products, but they are not characteristic of cancer. Dr. Hannover has not as yet observed cancer-cells in the third form of cancerous degeneration, i. e. in cancer alveolaris.

After these general remarks, our author proceeds to describe his first species, cancer medullaris, the encephaloid cancer of Dr. Walshe and others; but we omit the well known anatomical description of this morbid alteration, to arrive at the author's views respecting the process of its softening and destruction. This process of softening, which, as we have already pointed out, is accomplished by the destruction of the cancer-cells, takes place in two ways in the medullary species. 1. The centre, and shortly afterwards the great mass of the diseased growth breaks down, and forms a soft, semifluid pultaceous matter, which becomes daily thinner in consistence, so that at length the central contents may almost resemble serum in fluidity, while the external portions are still, to a certain extent, hard and resistant, and, from the admixture of blood with the fluid within, the whole contents become of a dark brown or chocolate hue, mingled with detached pieces of the cancerous mass.

2. Softening may commence in many points at one and the same time, and in such cases, a number of small white specks show themselves, consisting of a white pultaceous matter, composed of altered cells; these points or specks increase in size, and may run together into large cavities or canals, filled with the pultaceous matter above alluded to. Dr. Hannover has often met with this form of softening where the ground colour of the diseased mass was grayish, where its consistence was diminished, and it was semi-transparent; the white points then are very plainly seen upon the grayer ground, they are usually very numerous, and form sometimes curved lines (Rader) or streaks, upon the cut surface, so that at first sight, the mass may be regarded as appertaining to schirrous cancer.

Dr. Hannover does not venture to decide upon the immediate cause of the softening of cancer, but he thinks it possible that it is preceded by a process akin to that of inflammation if not absolutely identical with it, for he has (like Dr. Walshe) frequently observed exudation-corpuscles in the softened mass. It is, however, clear that, as Dr. Walshe has shown the distinct evidences of inflammation are not to be detected in many softened particles of cancer, inflammation cannot be—or at least cannot, in the present state of knowledge, be admitted to be—the sole agency at work in producing softening. Our countryman thinks, and we believe with much argumentative evidence in his favour, that the process must in some cases be analogous to the well-known *self-softening* (if it may be so styled) of fibrinous coagula.

With the historico-critical description of this first variety of cancer, we need not detain our readers, as our subject is solely to acquaint them with Dr. Hannover's original investigations. With the opinions of almost all writers up to the date of his own essay, our author seems to be thoroughly conversant, and it is gratifying to our insular self-esteem to find English authors so repeatedly and copiously quoted by our Danish brethren. Müller's cancer reticulare Dr. Hannover refers to the medullary species, and the reticular configuration which Müller considers to be its chief characteristic, our author asserts to result solely from the softening of the masses by the destruction of their cancer cells; in the latter point his experience agrees with that of Dr. Walshe. The second species of cancer, the scirrhus of authors, is described by Dr. Hannover, under the name of cancer schirrosus. Cellular tissue is a much more predominant constituent here than in the former species, rendering the mass much harder and more tough and resistant.

When cut into it exhibits a well-defined double composition, viz. of cancer-cells congregated into an almost gelatinous, white, or reddish, semi-transparent, or opaque mass, and these are contained in the intervals between the strong meshes of cellular membrane which traverse the tumour in every direction. Frequently the cellular membrane may be found aggregated in close bundles, which often spring from the point to which the cancerous growth is attached. Here the cancer-cells are generally but few in number, and upon their relative proportion to the cellular membrane, depends the variable consistence and aspect of the whole mass.

The softening of schirrous cancer progresses in the same manner as described in regard to the former species, viz. by destruction of the cancer-cells, but the cellular tissue participates only at a later period. When the mass has lost much of its pristine hardness, small cavities are often found within it, filled with a brownish ichorous fluid, in which Dr. Hannover frequently observed exceedingly minute crystals, of a character very difficult to determine, some of which, however, seemed to him to be prismatic. Our author considers the carcinoma fasciculatum of Müller to be merely a variety of this species. Dr. Walshe, on the other hand, having described specimens of this structure which fell under his notice, gives his opinion that it must be ranked as "a distinct and important variety of encephaloid." Time will settle the difference: *softness* is at all events one of the most remarkable characters of this cancer.

The second division of Dr. Hannover's essay (devoted to the consideration of cancer as it affects various parts of the human body) need not delay us: it contains nothing new.

In the third division of this essay, our author explains his own views of the nature and origin of cancer, and criticises with much fairness and candour, the opinions of others. These observations are here restricted to the two first-named species of cancer.

The origin of cancer from irritation or inflammation, as was maintained by Breschêt, Ferrus, and Broussais, is not now recognised by many pathologists, and we would gladly, had our space permitted it, have extracted Dr. Hannover's lucid account of the difference between cancer and the products of inflammation. This difference we think our author establishes in the most satisfactory manner, and we next find him opposing the doctrines of Cruveilhier and others, who maintain that the circulating system and, especially, the nervous system, have a great share in the original development and extension of cancer. Dr. Hannover rejects in toto the doctrine that the veins are the seat, i. e. the anatomical element affected in cancer. We have not space here to follow him through the six or eight pages he devotes to the consideration of this theory, and which has not found greater favour with our countryman Dr. Walshe. Dr. Hannover does not deny the occasional appearance of cancerous matter in the veins and sinuses, but he contends that this should not be regarded as conclusive evidence in favour of the venous system being its original seat. In fine, he adopts to a great extent the doctrines of Schleiden and of Schwann, as applied to cancerous growths. Müller and others have stated that they have not discovered any peculiar elementary form in cancerous tissue to serve as characteristic of their nature, but Dr. Hannover repeats the assertion, that he has never, save in cancerous growths, observed the peculiar cancer-cells, described in the first part of this essay. We have already noticed that our author is unwilling to speak decidedly as to the nature of cancer alveolaris, he states that he has recently had but two opportunities of examining this form of cancer; and in both instances, his examination, from causes he could not control, was more or less incomplete. However, he observes, that it is a remarkable fact, that both Gluge and Müller have discovered peculiarities under the microscope in this variety, which will, if further corroborated, entirely separate it from the medullary and schirrous forms. Nor has Dr. Hannover ever seen or read of a case where alveolar cancer, when extirpated by the knife, has been succeeded by the medullary species, an occurrence not altogether uncommon in the history of schirrous cancer. Indeed our author would wish to form two distinct genera of malignant disease, i. e. colloid and cancer, but from the insufficient data he has hitherto collected, he dares not positively separate them.

With Dr. Walshe then, Dr. Hannover evidently believes that cancer is formed *external* to the blood-vessels; that it originates in nuclei formed in the plastic exudation of the blood, and that these subsequently form cells, some of which are afterwards developed into fibres, in the shape of cellular tissue, while others, which have not this property of originating fibres, are the true cancer-cells. These last, like the cells of pus, are always the most rapidly productive when their nuclei are most numerous: thus in cancer medullaris we find many nuclei, and it is precisely in this species that we observe the most rapid development of the morbid growth, particularly when the skin has given way, and the pressure upon the cells has been thus partially removed. Cancer schirrosus increases more slowly; for many years it may remain stationary, and is less liable to be reproduced when once extirpated.

Finally, Dr. Hannover touches briefly on the disputed question of the constitutional character of cancer, and he is evidently not disposed to admit of it to the full extent that is conceded by many writers. Indeed, he states that cancer, when it remains long stationary in only one part of the body, has no positive title (why then in his definition admit that the disease depends on a diathesis?) to be considered a constitutional disease. Dr. Walshe, we know, has taught a doctrine the very reverse of this, and upon the review of a mass of facts and arguments, as extensive, perhaps, as was ever adduced in illustration of any medical question, comes to this conclusion: "*A cancerous tumour, under all circumstances, even should it remain single and stationary for years, is but the local evidence of a general vitiation of the system.*" It occurs to us inquire of Dr. Hannover how it happens, if his notion be correct, that when a perfectly quiet and old standing cancer is cut away from a person in *apparently* good general health, it sometimes (too often as surgeons know) happens that multitudes of cancers almost immediately spring up into activity in certain of the internal organs?

Our readers will have observed ere this that the researches of Dr. Hannover agree in most of their material points with the previous* and likewise the more recent investigations of Dr. Walshe. The essay of the former, is of course a much less elaborate production than the volume of the latter, but it is most satisfactory to find so close a coincidence between independent observations made by these two distinguished pathologists at nearly the same time in different parts of Europe. Perhaps after meeting with the work of Dr. Walshe, Dr. Hannover may favour us with a more ample development of his own views, aided by accumulated observations and increased experience, but to whatever subject in the whole range of physiology or pathology he may turn his attentive and inquiring mind, we are quite sure that his investigations will receive that notice and appreciation they have hitherto so eminently deserved.

* It is just to both parties to state, that Dr. Hannover was acquainted with, and has liberally availed himself of, Dr. Walshe's article, "Cancer," in the Cyclopædia of Surgery, published in 1840.

